# An Epigenetic Alphabet of Crop Adaptation to Climate Change

**DOI:** 10.3389/fgene.2022.818727

**Published:** 2022-02-16

**Authors:** Francesco Guarino, Angela Cicatelli, Stefano Castiglione, Dolores R. Agius, Gul Ebru Orhun, Sotirios Fragkostefanakis, Julie Leclercq, Judit Dobránszki, Eirini Kaiserli, Michal Lieberman-Lazarovich, Merike Sõmera, Cecilia Sarmiento, Cristina Vettori, Donatella Paffetti, Anna M. G. Poma, Panagiotis N. Moschou, Mateo Gašparović, Sanaz Yousefi, Chiara Vergata, Margot M. J. Berger, Philippe Gallusci, Dragana Miladinović, Federico Martinelli

**Affiliations:** ^1^ Dipartimento di Chimica e Biologia “A. Zambelli”, Università Degli Studi di Salerno, Salerno, Italy; ^2^ Centre of Molecular Medicine and Biobanking, University of Malta, Msida, Malta; ^3^ Bayramic Vocational College, Canakkale Onsekiz Mart University, Canakkale, Turkey; ^4^ Department of Molecular and Cell Biology of Plants, Goethe University, Frankfurt, Germany; ^5^ CIRAD, UMR AGAP, Montpellier, France; ^6^ AGAP, Univ Montpellier, CIRAD, INRA, Institut Agro, Montpellier, France; ^7^ Centre for Agricultural Genomics and Biotechnology, FAFSEM, University of Debrecen, Debrecen, Hungary; ^8^ Institute of Molecular, Cell and Systems Biology, College of Medical, Veterinary and Life Sciences, University of Glasgow, Glasgow, United Kingdom; ^9^ Plant Sciences Institute, Agricultural Research Organization Volcani Center, Rishon LeZion, Israel; ^10^ Department of Chemistry and Biotechnology, Tallinn University of Technology, Tallinn, Estonia; ^11^ Institute of Biosciences and Bioresources (IBBR), National Research Council (CNR), Sesto Fiorentino, Italy; ^12^ Department of Agriculture, Food, Environment and Forestry (DAGRI), University of Florence, Florence, Italy; ^13^ Department of Clinical Medicine, Public Health, Life and Environmental Sciences, University of L’Aquila, Aquila, Italy; ^14^ Institute of Molecular Biology and Biotechnology, Foundation for Research and Technology—Hellas, Heraklion, Greece; ^15^ Department of Biology, University of Crete, Heraklion, Greece; ^16^ Department of Plant Biology, Uppsala BioCenter, Swedish University of Agricultural Sciences and Linnean Center for Plant Biology, Uppsala, Sweden; ^17^ Chair of Photogrammetry and Remote Sensing, Faculty of Geodesy, University of Zagreb, Zagreb, Croatia; ^18^ Department of Horticultural Science, Bu-Ali Sina University, Hamedan, Iran; ^19^ Department of Biology, University of Florence, Sesto Fiorentino, Italy; ^20^ UMR Ecophysiologie et Génomique Fonctionnelle de la Vigne, Université de Bordeaux, INRAE, Bordeaux Science Agro, Bordeaux, France; ^21^ Institute of Field and Vegetable Crops, National Institute of Republic of Serbia, Novi Sad, Serbia

**Keywords:** abiotic stresses, adaptation, climate change, epigenetics, environmental stresses, epigenetic code

## Abstract

Crop adaptation to climate change is in a part attributed to epigenetic mechanisms which are related to response to abiotic and biotic stresses. Although recent studies increased our knowledge on the nature of these mechanisms, epigenetics remains under-investigated and still poorly understood in many, especially non-model, plants, Epigenetic modifications are traditionally divided into two main groups, DNA methylation and histone modifications that lead to chromatin remodeling and the regulation of genome functioning. In this review, we outline the most recent and interesting findings on crop epigenetic responses to the environmental cues that are most relevant to climate change. In addition, we discuss a speculative point of view, in which we try to decipher the “epigenetic alphabet” that underlies crop adaptation mechanisms to climate change. The understanding of these mechanisms will pave the way to new strategies to design and implement the next generation of cultivars with a broad range of tolerance/resistance to stresses as well as balanced agronomic traits, with a limited loss of (epi)genetic variability.

## 1 Epigenetics – Beyond the Classic Genetic Alphabet

The term “epigenetics” derives from “epigenesis,” coined by the physician and physiologist William Harvey at around 1650, for the conception of development as a gradual process of increasing complexity from initially homogeneous material present in the egg of different animals. This idea was originally proposed by Aristotle (Van Speybroeck et al., 2002). However, this concept deeply changed over time, and in 1942 the embryologist Conrad Waddington introduced the term “epigenetics” into modern biology defining it as “the whole complex of developmental processes” that lies between “genotype and phenotype” (Waddington, 1942).

In recent years, our understanding of the role of epigenetic mediated responses to environmental stimuli, especially to stresses, has greatly improved (Mladenov et al., 2021). Environmental stress factors, due to climate change, affect plant growth and pose a growing threat to sustainable agriculture and food security (Altieri et al., 2015). These factors include intense drought periods, excessive rainfalls eventually causing flooding, extreme temperatures, and heat waves, among others (Schiermeier, 2018). Although the acute responses of crops to single stresses are considered individually and in single occurrence are extensively studied, stresses typically occur in a chronic or recurring way and mostly in a combined manner. Recent studies suggest that plants have “a stress memory” that is guiding, or supervising in a way, their adaptation to chronic, recurring, and combined environmental stresses (Walter et al., 2013). In general, irrespective of whether environmental stimuli are chronic or not (such as drought, hyperosmotic, salinity, heat, pathogens, etc.), they can induce diverse epigenetic mechanisms, where key genes, such as Dicer-like 4 (DCL4) and Retrotransposon-like 1 (RTL1), play an important role.

**FIGURE 1:**
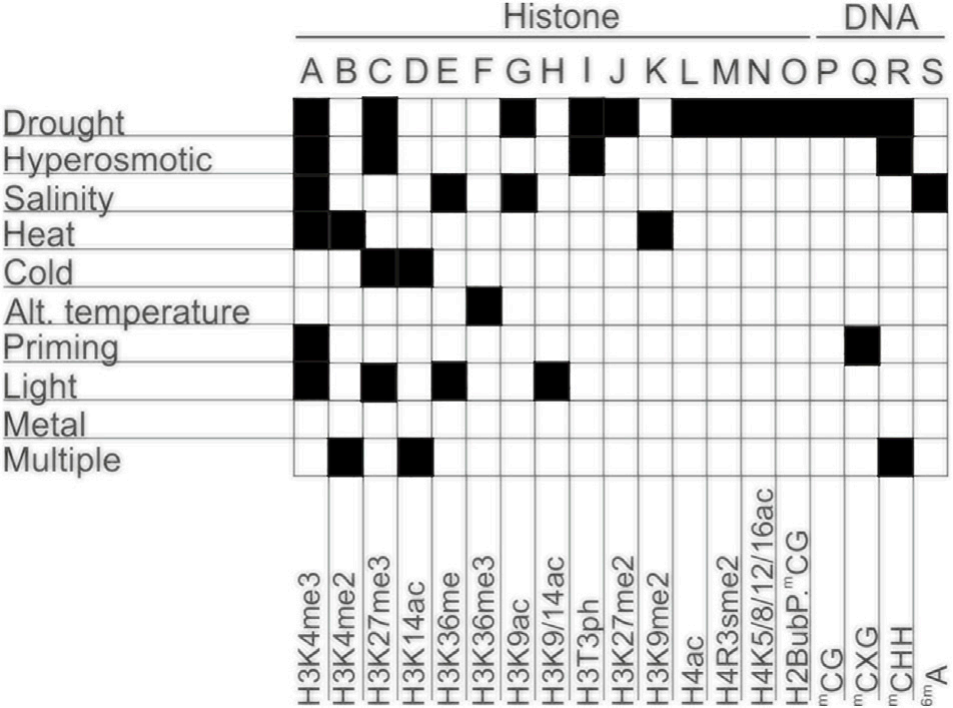
Deciphering the alphabet of epigenetic responses to the environmental stresses in plants. Different types of epigenetic modifications in response to different abiotic and biotic stresses. A-O—Histone modifications; P-R—Cytosine methylation; S—Adenine methylation.

Epigenetic mechanisms involved in plant responses to environmental stresses are not encoded by the classical four-letter genetic alphabet (Faltýnek et al., 2020). Hence, epigenetic modifications are usually chemically expressed by expanding the standard four-letter genetic alphabet by the addition of a special mark to a letter (nucleobases), thus creating a specific “epigenetic alphabet” (Figure 1).

### 1.1 From A to S—“Basic” Epigenetic Alphabet

#### 1.1.1 A-O: Histone Variants and Histone Post-Transcriptional Modifications

**FIGURE 2:**
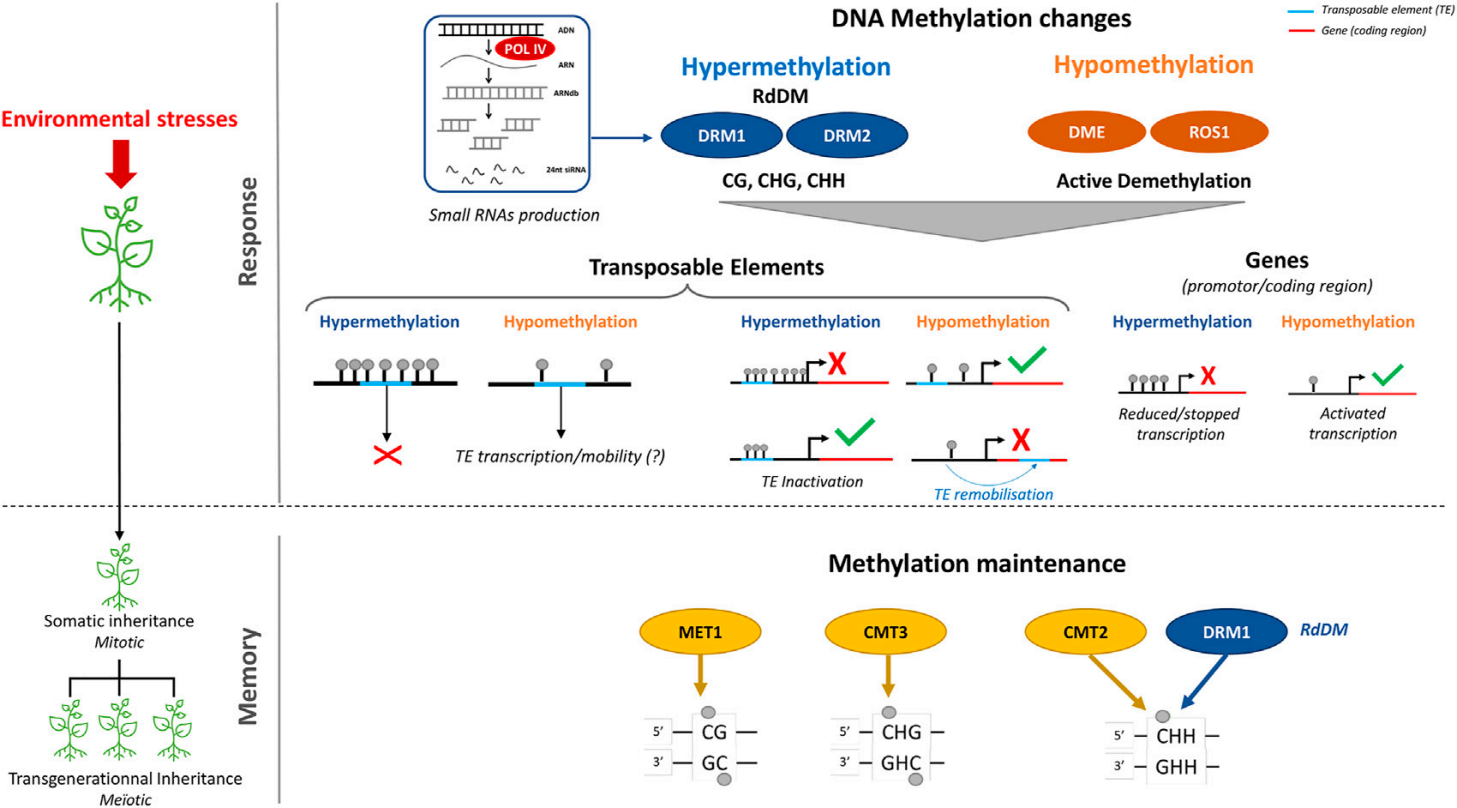
Roles of DNA-methylation in environmental stress responses and memories in plants. Changes in DNA-methylation landscape are part of the response of plants to environmental stresses. De novo methylation, which is targeted at specific loci by small-RNAs, is established by the RNA-dependent-DNA-Methylation pathway (RdDM) whereas, DNA demethylation at specific loci requires functional DNA Glycosylase Lyase also called DNA demethylase such as Repressor of Silencing 1 (ROS1). Modification of DNA methylation patterns at genes may result in changes in gene expression level leading to gene induction or repression. In addition, stress induced DNA methylation variations may occur at transposable elements (TEs) and determine their inactive or active state. When hypomethylated and transcriptionally active, TEs may indirectly influence the expression of genes located in their vicinity, whereas their hypermethylation has the reverse effect. Additionally, the mobility of TEs may generate new regulatory patterns or mutations leading to loss of gene function when their insertion occurs in genes. Maintenance of stress induced patterns of DNA methylation through cell division (Mitosis or meiosis), results in an epigenetic memory. This memory requires the context-specific DNA-methyltransferases METHYLTRANSFERASE-1 (MET1), CHROMOMETHYLASE-3 (CMT3) for CG and CHG sequence context, respectively. Methylation in the CHH sequence context is maintained by CMT2 or by the RdDM pathway in heterochromatic and euchromatic regions, respectively.

Chromatin structuring and remodeling, which are key regulatory processes for controlling the accessibility of genes to the transcriptional machinery, play an important role in plant responses to climate change (Song et al., 2021) (Figure 2).

The basic functional unit of the chromatin is the nucleosome, which consists of a histone octamer made of two copies of each of the histone H2A, H2B, H3, and H4 wound by 147 bp of DNA. The histone H1 binds to the “linker DNA” comprising 20–80 nucleotides that separate two nucleosomes (Annunziato, 2008). Canonical histones, except for H4, have minor variants which can be incorporated into the nucleosome throughout the cell cycle. Canonical histones and their variants differ only by a few amino acid residues, but their exchange in the nucleosome can modulate the exposure of DNA and regulation of transcription by directly influencing the chromatin structure. For example, the H2A.Z variant located in gene bodies ensures the repression of heat and osmotic stress-related genes in the absence of stress, while eviction of H2A.Z allows their transcriptional induction upon stress (Cortijo et al., 2017; Sura et al., 2017). Mutants of the SWR1-like chromatin remodeling complex which are impaired in H2A.Z installment show enhanced resistance to pathogens, highlighting H2A.Z importance for adaptive response to both abiotic and biotic stresses (March-Díaz et al., 2008). Another example is provided by the stress-inducible H1 variant H1.3 in *Arabidopsis thaliana*, which modulates stomata under non-stress or light and water-limited conditions (Rutowicz et al., 2015).

In addition, post-translational modifications of histones may lead to changes of chromatin structure and packaging and modify the accessibility of cis-regulatory elements to transcription factors and associated protein complexes (Zhang X. et al., 2020). Among the 26 histone post-translational modifications (HPTMs) described in the literature (Zhao et al., 2015), two have been intensively studied in the context of the response to stress, namely acetylation and methylation, while recent work suggest that ubiquitination and phosphorylation are also involved in this process. As all other HPTMs, these marks are established by histone writers complexes such as histone acetyltransferases (HAT), methyltransferases (HMT), kinases, and ubiquitinases, and removed by “erasers” including deacetylases (HDA), demethylases (HDM), phosphatases, and de-ubiquitinases (Xu et al., 2017; Maeji and Nishimura, 2018). Acetylation which occurs on lysine residues (K) on histones H3 and H4 respectively at positions 9, 14, 18, 23, and 27, and positions 5, 8, 12, 16, and 20, neutralizes the positive charge of histones thereby weakening the interaction between histones and DNA. In contrast, deacetylation has the opposite effect and results in chromatin condensation (Shahbazian and Grunstein, 2007; Prakash and Fournier, 2018). Consistently, histone acetylation has been associated with active gene expression (reviewed in Hu Y. et al., 2019). Several studies have demonstrated that the abundance and/or distribution of acetylated histones change in plants facing abiotic stresses or pathogen attacks (reviewed in Hu Y. et al., 2019; Lu et al., 2018; Park et al., 2018). Furthermore mutants affected in either of these enzymatic activities present altered responses to abiotic stresses (reviewed in Hu Y. et al., 2019). Among the HAT, several studies have shown that the GCN5 protein plays a central role in coordinating the response to heat and salt stress in *A. thaliana* (Hu et al., 2015; Zheng et al., 2018). Inversely, deacetylation which leads transcriptional repression affects the transcriptome landscape under abiotic stress conditions (Park et al., 2018).

The mono-, di- or tri-methylation of histone tails, which occurs at arginine (R) or lysine (K), alters the hydrophobicity of histone side chains thereby the interaction with reader proteins and the transcriptional machinery. However, R and K methylation has diverse effects on chromatin organization and gene expression depending on the position of the modified amino acid (Lämke and Bäurle, 2017). Asymmetric H4R3me2 (dimethylation (me2) of the third arginine (R3) of Histone 4 (H4), H3K4me3, H3K36me2/3 are associated with active transcription, while symmetric H4R3me2, H3K9me2/3, and H3K27me3, that exist symmetrically on the two copies of identical histones in the same nucleosome, correlate with transcriptional silencing (Bobadilla and Berr, 2016; Ueda and Seki, 2020). Numerous works that either analyzed the dynamics of histone methylation or the reponse of mutants affected in HMT aor HDMT activities have now shown the importance of histone methylation in the development and responses to stresses (reviewed in Ueda and Seki, 2020). Importantly, data suggest that the removal of repressive methylation marks is necessary for some stresses to unlock the expression of stress related genes (Shen Y. et al., 2014; Huang et al., 2019b).

In contrast to acetylation and methylation, histone ubiquitination and phosphorylation have been only sparsely studied. Monoubiquitination of histone lysine H2Bub is considered an active mark in salt and drought stress response (Chen et al., 2020). However, the mono-ubiquitination of histones is an important HPTM that occurs on histones H2A and H2B at lysine K121 and K143 respectively (March and Farrona, 2018). Whereas H2B mono-ubiquitination (H2B ub) marks active genes in association with methylation at K4 and K36 of histone H3, H2Aub, which is established by the PRC1 (Polycomb Repressive Complex 1) upon recruitment at H3K27me3 marks established by the PRC2, maintain the chromatin in a closed state and is associated with the repression of gene expression (March and Farrona, 2018). It is only recently that a possible role H2Bub in the response to drought stress was established in cotton (Chen et al., 2019). and in *A. thaliana* and rice, respectively, by regulating cutin biosynthesis (Ménard et al., 2014; Patwari et al., 2019) and ABA signaling (Ma et al., 2019).

Finally, the phosphorylation of histone H3 which can occur on threonine and arginine has been essentially studied in the context of the cell cycle (Houben et al., 2007) and its putative role in stress responses is not well understood so far. However, phosphorylation at H3T3 is increased in pericentromeric regions under drought conditions to repress transcription, acting in an antagonistic manner to H3K4me3 (Wang Z. et al., 2015).

In addition to the response to stresses, HPTMs have also been implicated in stress memory such as histone methylation which can be maintained for a relatively long period in primed plants (Lämke, and Bäurle, 2017).

#### 1.1.2 P-S: DNA Methylation

**FIGURE 3:**
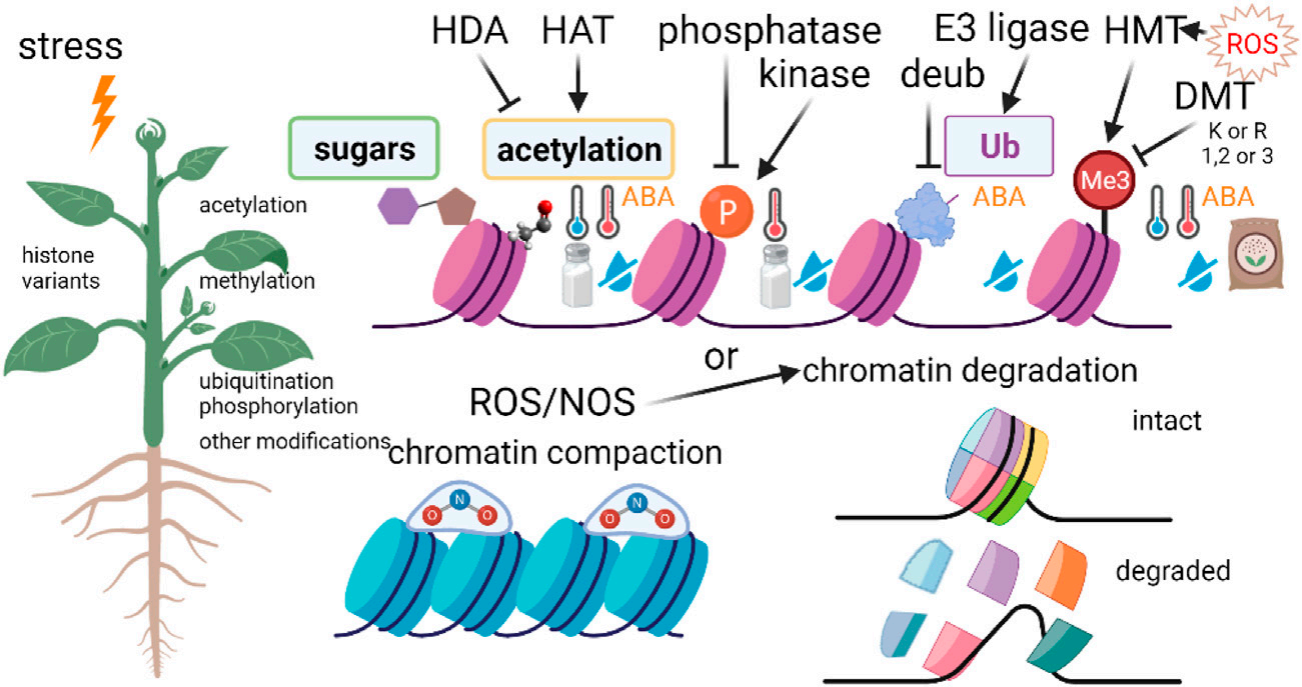
Histone modifications in response to environmental stresses. HAT, histone acetyltransferase; HDA, histone deacetylase; HMT, histone methyltransferase; ROS, reactive oxygen species; ABA, abscisic acid.

DNA methylation plays an important role in the regulation of gene expression and plant reaction to environmental stresses (Kumar and Mohapatra, 2021a) (Figure 3), In plants, DNA methylation predominantly occurs by the addition of a methyl group to the fifth position of the pyrimidine ring of cytosine bases or the sixth position of the purine ring of adenine bases, which is referred to as 5-methylcytosine [5 mC] or N^6^-methyladenine [6 mA] DNA methylation, respectively (Liu and He, 2020).

##### 1.1.2.1 Cytosine Methylation

Cytosine methylation in plants occurs in the two symmetrical q sequences contexts CG, and CHG, and in the non-symmetrical one CHH (where H is A, C, or T). Cytosine methylation is high at heterochromatic regions (centromeres, transposable elements (TE), other repetitive elements) is involved in their transcriptional silencing (Transcriptional Gene Silencing, TGS). In contrast, methylation levels are low in euchromatic regions (Liu and He, 2020).

Mechanisms involved in the regulation of DNA methylation depend on the sequence contexts and therefore occur following three different mechanisms. In the CG context, the DNA methyl transferase (DNMT) MET1 together with additional cofactors including VARIANT IN METHYLATION (VIM), and decrease IN DNA METHYLATION 1 (DDM1) methylate the unmethylated cytosine incorporated during DNA replication in the newly synthesized DNA strand (Law and Jacobsen, 2010), whereas the chromomethylase 3 (CMT3) and to a lower extend CMT2 will fulfill a similar function at CHG motives (Du et al., 2012). Methylation maintenance at CHH asymmetrical motives requires reinstalling methylation at the newly synthesized unmethylated DNA strand by the Domain Rearranged methyltransferase 2 (DRM2) and the RNA-dependent DNA methylation (RdDM) in euchromatic regions, or CMT2 methyltransferase in heterochromatic regions enriched in histone H1 where the RdDM pathway is inhibited (Zemach et al., 2013; Zhang H. et al., 2018). *De novo* methylation that occurs at non-methylated sites in any sequence context is mediated through the RdDM pathway, and requires small interfering RNAs (siRNAs), scaffold RNAs and several additional proteins (Zhang H. et al., 2018).

In addition, active DNA demethylation is gaining significant attention because it is involved in many biological processes in plants, and in the response to various stresses (Liu and Lang, 2020). 5 mC can be either passively removed by simple dilution after DNA replication, or actively eliminated by specific enzymes, namely the 5-methylcytosine glycosylase-lyase that belongs to the DEMETER (DME)/REPRESSOR OF SILENCING (ROS1) family in *A, thaliana* and DEMETER-LIKE (DML). These DMLs are bi-functional enzymes exercising the 5 mC creating an abasic site, likely repaired by unknown DNA polymerases and ligases activities. The whole process results in a net loss of cytosine methylation (Law and Jacobsen, 2010).

Variations in DNA methylation can occur during inbreeding, plant aging, and in the response to different stresses (Quadrana and Colot, 2016; Zhang Q. et al., 2018). These variations may underlay phenotypic variation (Noshay and Springer, 2021) as demonstrated by analyzing Epigenetic Inbred Lines (EpiRILs) in *A, thaliana* (Quadrana and Colot, 2016). In addition, EpiRILs allowed demonstrating that differences in the epigenetic landscape of plants can lead to a significant plastic response to stresses (Kooke et al., 2015), as DNA methylation changes were observed in stressed plants (reviewed in Zhang Q. et al., 2018). In addition, impairing enzymes involved in DNA methylation leads to variable survival in response to stresses, highlighting the fundamental role of DNA methylation in the plant responses to stresses (Yao et al., 2012; Shen X. et al., 2014; Wibowo et al., 2016).

##### 1.1.2.2 Adenine Methylation

The recent discovery that adenine can also be methylated although at very low rates, add another layer of complexity to the epigenetic processes affecting plant genomic DNA (reviewed in Chachar et al., 2021). As for cytosine methylation, 6 mA DNA methyltransferases have been identified, as well as associated demethylases (Chachar et al., 2021). Interestingly, 6 mA associates with active gene expression, which contrasts with the main function associated with cytosine methylation (Zhang Q. et al., 2018; Liang et al., 2018). Recent evidence also suggests that rice plants with various levels of 6 mA present variable responses under different abiotic stresses, suggesting a potential role of 6 mA in the plant responses to environmental stimuli (Zhang Q. et al., 2018).

6 mA is directly involved in heterochromatin regulation in mouse embryonic cells. It also participates in the regulation of mRNA encoding HAT or HMT, and is involved in the recruitment of histone modifiers during transcription thereby affecting the deposition of specific epigenetic marks in animals (reviewed in Kan et al., 2021). However, further studies are needed to elucidate if 6 mA has similar roles in plants.

### 1.2 From T to Z?—RNA-Mediated Epigenetic Modifications

The regulation of gene expression in response to stresses, both at the transcriptional and post-transcriptional levels, confers plasticity, and adaptability to plants (Wang et al., 2017; Song X. et al., 2019). This regulation is induced by small and long non-coding RNAs (sRNAs 20–24 nt, and lncRNAs > 200 nt), thus adding new letters to the “epigenetic alphabet,” beyond ones created by histone modifications and DNA methylation (Figure 1).

Non-coding RNA may be involved in the constitutive repression of transposon elements. MicroRNAs (miRNAs) are essential factors able to determine the specificity of post-transcriptional regulations. They originate from the cleavage of endogenous transcripts of miRNA (MIR) genes by DICER-LIKE 1 (DCL1). Loaded into AGO1, miRNAs regulate the gene expression by degradation or translational repression of target mRNAs. Although miRNAs are primarily involved in the PTGS regulation of gene expression, recent evidence suggests that they may also participate in epigenetic pathways, although indirectly. For example, modulation of miRNA populations was suggested to shape the epigenetic memory of stresses by modulating the expression of epigenetic regulators in Norway spruce (Yakovlev and Fossdal, 2017). In addition, miRNA may play direct although minor roles in DNA methylation through the non-canonical RdDM pathway (Cuerda-Gil and Slotkin, 2016), including in response to environmental stimuli (Iwasaki et al., 2019).

Small interfering RNAs (siRNAs) arise from the processing of intermediate double-stranded RNAs synthesized by RNA-dependent RNA polymerases (RDRs) (Song X. et al., 2019). Among them, TE-derived siRNAs are produced upon transcription and/or transpositional reactivation of TEs in response to stress (Hou et al., 2019). The plant-specific RNA polymerase IV generates single-stranded siRNA precursors, converted into double-stranded RNAs (dsRNAs) by RDR2. These dsRNAs are processed by DCL3 for producing 24-nt mature siRNA and loaded preferentially into AGO4 (Law and Jacobsen, 2010). At the same loci, another plant-specific RNA polymerase V generates non-coding transcripts allowing the recruitment of the siRNA-AGO4 complex through sequence complementarity, as well as DRM2 (Du et al., 2015). Consequently, *de novo* DNA methylation occurs at different loci (Erdmann and Picard, 2020) in all cytosine sequence contexts (Law and Jacobsen, 2010). Distinct Dicer-type nucleases are involved in miRNAs/siRNAs production. They are subsequently recruited by distinct proteins of the AGO family (Iki, 2017), which act together within the miRNA-induced silencing complex to target complementary sequences of coding and non-coding RNAs (Song X. et al., 2019).

LncRNAs regulate gene expression at the epigenetic, transcriptional, post-transcriptional, translational, and post-translational levels (Sun et al., 2018; Zhang et al., 2019; Wu et al., 2020). LncRNAs are transcribed by RNA polymerase II, III, IV, and V, and have specific spatial structures and spatiotemporal expression patterns. They are divided into five categories according to their position in the genome, next to or far from protein-coding genes: sense, antisense, bidirectional, intronic (incRNA), and large intergenic lncRNA. Many plant lncRNAs are differentially expressed by abiotic and biotic stresses (Wang et al., 2018; Yin et al., 2019) and were suggested to play an important role in this context (Urquiaga et al., 2021).

Recent technical advances have revealed widespread and sparse modification of mRNAs, providing an additional layer of complexity to the regulation of gene expression. Prevalent mRNA modifications, namely the N^6^-methyladenosine (m6A) and 5-methylcytidine (m5C), are modulated by specific writers (RNA methyltransferase, e.g., AlkB), readers, and erasers (RNA demethylase). The writer complex, also known as “methylosome,” adds m6A at conserved sites and comprises a catalytic heterodimer METTL3/METTL14; MTA in *A. thaliana*, associated with the regulatory proteins FIP37 (FKBP12 INTERACTING PROTEIN 37)) and VIRILIZER. The corresponding mutants are embryo lethal (Zhong et al., 2008; Shen et al., 2016; Růžička et al., 2017). Furthermore, m6A stabilizes transcripts required for salt and osmotic stress response (Anderson et al., 2018), suggesting roles for m6A beyond development. Polymethylated mRNAs (i.e. carrying many m6A modifications) facilitate inter/intramolecular interactions, a property referred to as “multivalency.” Multivalency enables m6A mRNAs to participate in assemblies comprising proteins, RNAs, and metabolites called “biomolecular condensates” due to their capacity to concentrate molecules. Condensate formation may rely on liquid-liquid phase separation (LLPS), whereby a solution de-mixes into two or more distinct phases (Huang et al., 2021). Proteins with intrinsically disordered regions (IDR) in many cases can promote LLPS. For example, the IDR-enriched YTH domain proteins EVOLUTIONARILY CONSERVED C-TERMINAL REGION2/3/4 (ECT2/3/4), which can read m6A, modulate leaf development and localize in the biomolecular condensates known as stress granules (SGs) (Kosmacz et al., 2019). Yet, there is a lack of understanding of ECT functions in the SGs. SGs form rapidly upon stress onset to readjust the transcriptome by degrading or storing mRNAs and thus optimizing adaptation (Gutierrez-Beltran et al., 2015). As has been shown for animals, ECTs may regulate SGs formation during stress (Fu and Zhuang, 2020), thereby adjusting the transcriptome landscape indirectly by recruiting m6A-modified RNA molecules in SGs, whereby they are kept inert.

Several studies suggest that the m6A writers AlkB homologs (ALKHB) regulate stress responses due to their gene expression levels modulation upon stress (Hu H. et al., 2019). *A. thaliana* has 13 ALKHB proteins, and ALKBH9B demethylates m6A and affects viral spread (Martínez-Pérez et al., 2017), while ALKBH10B influences flowering by controlling SQUAMOSA PROMOTER-BINDING PROTEIN-LIKE (SPL) 3, SPL9, and FLOWERING LOCUS T mRNA levels (Duan et al., 2017). This link is indirect and merits further investigation.

Apart from m6A, we know little about other mRNA modifications in plants. Recent evidence suggests a link between m5C and RNA mobility. Mobile mRNAs are rich in m5C (Yang et al., 2019). Yet, the molecular machinery involved in recognizing and distributing m5C mRNA is still unknown. Furthermore, there is evidence, mainly from animal systems, that mRNA modification also plays a direct role in epigenetics (Kumar and Mohapatra, 2021b; Kan et al., 2021). Whether similar roles for epi-modification of plant mRNAs exist in plants requires further investigation.

## 2 Epigenetic Alphabet—(De)coding the Stress Response

Climate change is altering the environments in which all organisms develop and thrive. Plant species, as sessile organisms, can adjust to these novel conditions through phenotypic plasticity, natural selection and eventually can change habitat to follow their optimal growing conditions, these possibilities being not mutually exclusive. Epigenetic modifications that occur in plants are also part of their response to changes in their environment. Those epigenetic changes are adding to natural mutations, with epigenetic marks creating an enlarged version of the genetic alphabet, thus increasing the variety of phenotypes within the stress-affected habitat (Faltýnek et al., 2020). When heritable to the progeny, they become a certain kind of “norm,” enabling us to further decode stress response in crop of interest and apply it for resilience improvement.

In the context of stress responses, these histone-modifying complexes are directed by stress-induced transcription factors to their appropriate targets. For example, the COMPASS H3K4 methyltransferase complex is recruited by bZIP transcription factors and brings about methylation of H3K4 (Song et al., 2015). Furthermore, among the numerous messengers, such as calcium, redox signaling, membrane integrity, G-proteins, mitogen-activated protein kinases (MAPKs), plant stress hormones (salicylic, jasmonic and abscisic acid, ethylene) that modulate the response of plants to stresses, the Reactive Oxygen Species (ROS) and the Reactive Nitrogen Species (RNS) have received increasing attention over the last decade as they are key players of the integrated responses of plants to these stresses, in addition to their fundamental functions in plant development (Sewelam et al., 2016; Huang et al., 2019a; Kumar et al., 2020). Indeed, the plant responses to different abiotic stresses, such as heat, chilling, excessive light, drought, ozone exposure, UV-B irradiation, osmotic shock, heavy metals, and organic pollutants involves a rapid oxidative burst that leads to the generation and/or accumulation of oxidants such as ROS and RNS. These reactive species are essential signaling systems that participate to multiple processes, necessary to adjust the metabolism or physiology either at the whole plant or tissue level or in a specific subcellular compartment (Waszczak et al., 2018).

Redox intermediates play also a critical role in the regulation of epigenetic mechanisms in response to plant stresses. They govern DNA methylation levels: increases in ROS caused DNA hypomethylation both in tobacco (Choi and Sano, 2007; Poborilova et al., 2015) and pea (Berglund et al., 2017). Similarly, in rice, RNS caused a heritable hypomethylation (Ou et al., 2015). In addition, redox intermediates often regulate enzymes involved in histone methylation and acetylation (Ojima et al., 2012). In maize, ROS, generated by heat stress, induced histone hyperacetylation (Wang P. et al., 2015). Heritable changes induced by the environment have been shown in *Linum usitatissimum* L. (Cullis, 1986), in *Mesembryanthemum crystallinum* L. (Bohnert et al., 1995), and *Brassica nigra* L. (Waters and Schaal, 1996). Since the ‘90s hypermethylation of heterochromatic loci has been reported in tobacco, either in response to osmotic stress (Kovar˘ik et al., 1997) or in silenced genes in transgenic plants (Meyer et al., 1992; Meyer and Heidmann, 1994). On the contrary, hypomethylation has been documented in chicory root tips (Demeulemeester et al., 1999) and *A. thaliana* (Finnegan et al., 1998) when exposed to low temperature. Epigenetic changes have been observed in tissue cultures, while methylation polymorphisms have been frequently observed during the propagation of tissue cultures at the level of repeated sequence (Smulders et al., 1995) and may contribute to somaclonal variation (Kaeppler et al., 2000). All these variations in genome methylation might be part of the plant’s adaptation mechanisms to abiotic stresses (Martienssen and Richards, 1995; Kovarík et al., 1997).

**FIGURE 4:**
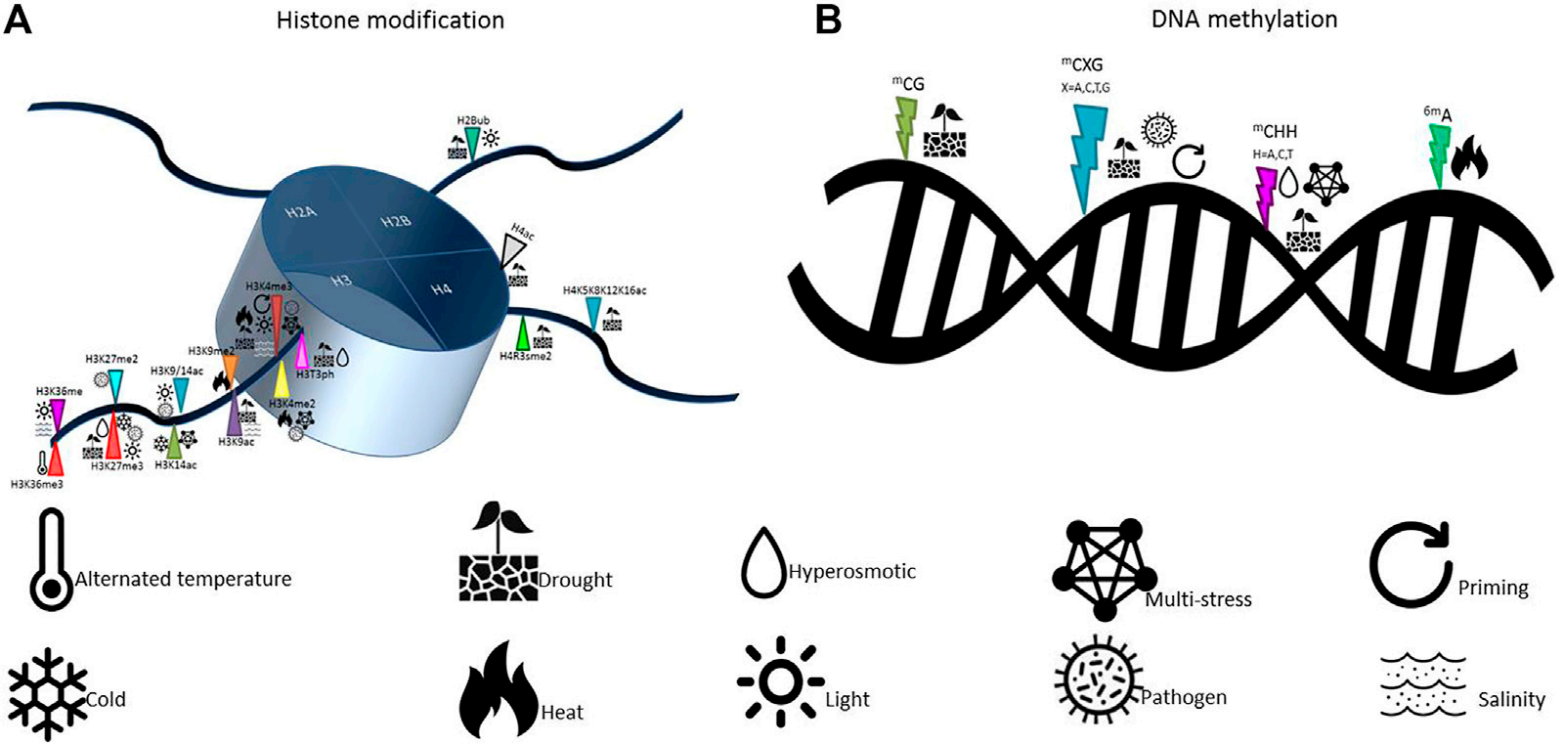
Epigenetic mechanisms involved in plant response to stress. Histone modifications **(A)** include acetylation/deacetyaltion and methylation/demethylation, while DNA methylation **(B)** includes cytosine methylation and adenine methylation processes.

Furthermore, under abiotic constraints, plants show multiple alterations in their sRNAome, leading to changes in the accumulation of individual sRNAs or through their specific induction in stress conditions, as shown in annual plants (Liu et al., 2017; Pan et al., 2017; He et al., 2019) and cultivated perennials plants (Leclercq et al., 2020). The respective proportions of the different sRNA classes may also be modified in response to stress, adjusting a genome-wide gene expression reprogramming to different sRNA-dependent regulation mechanisms. Few examples are known in plants responding to developmental or abiotic cues. A switch between 24 and 21 nt sRNA has been observed in rubber trees upon the occurrence of the stress-induced Tapping Panel Dryness syndrome (Gébelin et al., 2013), as well as in apple trees during the vegetative-to-floral transition with changes within 24 nt sRNA population (Guo et al., 2017). In cereals, different cultivars showed altered abundance in miRNAs contents which was associated with differences in stress sensitivity and in the modulation of a wide set of genes referable to drought tolerance (Bakhshi et al., 2017; Fard et al., 2017). A summary of the most recent epigenetic modifications, in response to different types of stresses, as well as the alphabet of epigenetic responses to the environmental stresses in plants are presented in Figures 1, 4; Table 1, respectively.

**TABLE 1:** Most recent examples of epigenetic modifications in plants in response to different types of abiotic stresses. Duration of epigenetic state, type of epigenetic modification, key proteins involved (M: mediators; T: their targets).

Species	Stress condition	Epigenetic state duration	Epigenetic and post-transcriptional modifications			Key proteins involved	References
			Histone modifications	DNA methylation	Non-coding RNA		
Drought/Osmotic							
*Arabidopsis thaliana*	Four cycles of 2 h dehydration/22 h rehydration	Up to 5 days	H3K4me3	—	—	T: RD29B, RAB18	Ding et al. (2012)
*Arabidopsis thaliana*	One or two cycles of 2 h dehydration/22 h rehydration	5 days	H3K4me3, H3K27me3	—	—	M: CLF	Liu et al. (2014)
*Arabidopsis thaliana*	1–8 h dehydration	Up to 5 h after rehydration from a 4 h stress	H3K9Ac	—	—	T: RD20, RD29A	Kim et al. (2012)
*Oryza sativa*	7–9 days without watering	ND^a^	—	—	mir162b	T: TRE1	Tian et al. (2015)
*Oryza sativa*	7–9 days without watering in seedlings or during panicle development	ND	—	—	miR164	T: NACs	Fang et al. (2014)
*Arabidopsis thaliana*	−0.5 MPa PEG for 10 days	ND	—	—	miR393	T: TIR1, AFB2	Chen et al. (2012)
*Arabidopsis thaliana*	12 days without watering	12 days	H4R3sme2	—	—	M: CAU1/PRMT5/SKB1	Fu et al. (2013)
						T: CAS	
*Arabidopsis thaliana*	30% PEG for 7 days	7 days	H3T3ph	—	—	M: MLK1/2	Wang et al. (2015a)
						T: pericentromeric regions	
*Arabidopsis thaliana*	14 days without watering	14 days	H4Ac	—	—	T: PDC, ALDH2B7	Kim et al. (2017)
*Arabidopsis thaliana*	7 days without watering	14 days	H3K4me3	—	—	M: ATX4, ATX5	Liu et al. (2018)
						T: AHG3	
*Gossypium hirsutum*	7 days in MS medium with up to 40% PEG	12 days	H2Bub	—	—	M: AtHUB2	Chen et al. (2019)
						T: GhDREB	
			H3K4me3				
*Arabidopsis thaliana*	14 days without watering	14 days	H3Kac, H4ac	—	—	M: HDA15, MYB96	Lee and Seo, (2019)
						T: RHO gtpase	
*Popolus trichocarpa*	7 days without watering	7 days	H3K9ac	—	—	M: AREB1-ADA2b-GCN5	Li et al., 2019
						T: PtrNAC006, PtrNAC007, PtrNAC120	
*Oryza sativa*	5–7 days without watering	5–7 days	H2Bub1	—	—	M: OsHUB2, OsOTLD1	Ma et al. (2019)
						T: MODD	
*Zea mays*	Water content threshold of 25% of the available water for 10 days	7 days	H3K4me3, H3K9ac	—	—	T: ZEP1, NCAD6, AP2/EREBP, NAC, MADS4, MADS15	Forestan et al. (2020)
*Glycine max*	13 days without watering	—	—	—	miR169g	T: NFY	Ni et al. (2013)
*Oryza sativa*	7 days without watering	—	—	—	miR162b	T: TRE1	Tian et al. (2015)
*Arabidopsis thaliana*	11–15 days without watering	—	—	—	miR168a	M: AGO1	Li et al. (2012)
*Arabidopsis thaliana*	14 days without watering	—	—	—	miR396a, miR396b	T: GRF	Liu et al. (2009)
Hyperosmotic							
*Arabidopsis thaliana*	Priming with 50 mM NaCl, 10 days recovery, 14 days in 80 mM NaCl	10 days	H3K27me3	—	—	T: SOS5, LRP1, SCARECROW	Sani et al. (2013)
*Medicago truncatula*	204 mM NaCl for 1 week	ND	H3K4me2	mCHH			Yaish et al. (2018)
Salt							
*Arabidopsis thaliana*	Priming in 100 mM NaCl for 24 h, recovery for 48 h, 200 mM NaCl	5 days	H3K4me3	—	—	M: HY5	Feng et al. (2016)
						T: P5CS1	
*Arabidopsis thaliana*	200 mM NaCl for 6 h	ND	H4R3sme2	—	—	M: SKB1	Zhang et al. (2011)
						T: LSM4	
*Medicago sativa*	200 mM NaCl up to 24 h	ND	H3K9Ac	—	—	T: MsMYB4	Dong et al. (2020)
*Glycine max*	200 mM NaCl, 4 h	ND	HDAC	—	miR482bd-5	HEC1	Cadavid et al. (2020)
*Arabidopsis thaliana*	100–150 mM NaCl for 9 days	—	—	—	miR393	T: TIR1, AFB2	Chen et al. (2015)
Heat							
*Arabidopsis thaliana*	Acclimation: 37°C for 1 h, 23°C for 90 min, and 44°C for 45 min	3 days	H3K4me2	—	—	M: HsfA2	Lämke et al. (2016)
			H3K4me3			T: HSPs	
*Arabidopsis thaliana*	Acclimation: 37°C for 1 h, 23°C for 90 min, and 44°C for 45 min	3 days	Histone occupancy	—	—	M: FGT1	Brzezinka et al. (2016)
						T: HSPs	
*Brassica rapa*	42°C for 3 h per day for 7 days	Transgenerational	—	—	miR168	AGO1	Bilichak et al. (2015)
*Arabidopsis thaliana*	Basal: 44°C for 50 min	ND	—	—	TAS1 (tasiRNA)	HTT1/2	Li Shuxia et al. (2014)
	Acquired: 37°C for 1 h, 2 h 22°C, 44°C for 3.5 h	—					
*Arabidopsis thaliana*	37°C for 24 h	Transgenerational	—	—	siRNA	—	Ito et al. (2011)
*Arabidopsis thaliana*	37°C for 2 h	ND	—	—	miR408	CSD1, CSD2, GST-U25, CCS1, SAP12	Ma et al. (2015)

a ND, No data available.

### 2.1 Drought Stress

Water availability is one of the most important and prevalent environmental cues which affect the growth, development, and productivity of plants, and ultimately, their survival. Many known epigenetic regulations were shown to play a significant role in acclimation and adaptation to drought stress (Varotto et al., 2020) Concerning climatic change, one of the most important abiotic stresses, water scarcity, is becoming increasingly critical for the survival of plants and crop productivity and yield. Moreover, in long-lived perennial tree species, the water balance in the organ tissues is crucial for growth, survival, and reproductive capability, and influences their distribution along with the environment and climate gradient (Jenkins et al., 2018). Therefore, understanding how plants respond to water stress/scarcity would allow us to inform breeders to select new varieties more resilient to this kind of stress. Hence, many studies on epigenetic changes associated with water stress/scarcity or drought have occurred in recent years both in crops and woody species (Ashapkin et al., 2020; Varotto et al., 2020; Kapazoglou et al., 2021).

MYB96 transcription factor was identified in *A. thaliana* (Lee and Seo, 2019), and AREB1 in poplar as a HAT recruiter in response to drought stress, which affects the acetylation of the H3K9 and thereby the expression of NAC genes (Li et al., 2018). HDA6 and HDA15 regulate genes participating in the jasmonate signaling network and production of Rho of plants (RHO GTPases) by deacetylation of H3K914ac and H4K5K8K12K16ac, respectively, (Jiang et al., 2020). HDA9 is one of the major histone deacetylases, which regulates the expression of drought-responsive genes in *A. thaliana* (Lee and Seo, 2019). The increased expression of BdHD1 in *Brachypodium* caused lower acetylation of H3K9 affecting 230 genes and leading to an abscisic acid hypersensitive phenotype (Song J. et al., 2019). H3K4 methylation is widespread histone methylation in response to drought stress. In *A. thaliana*, the lower levels of H3K4me3 or H4R3sme2 cause increased drought stress tolerance (Liu et al., 2018), while H3T3ph, the phosphorylation of H3 at the threonine of the pericentromeric part, causes an increase in osmotic tolerance (Wang Z. et al., 2015). Monoubiquitination of H2A and H2B is also related to drought tolerance. In fact, H2Bub acts on changing abscisic acid signaling and wax biosynthesis and thereby enhancing drought tolerance not only in *A. thaliana* but in cotton and rice, as well (Chen et al., 2019).

In maize, the levels of H3K4me3, H3K27me3, and H3K9ac were investigated after exposure to prolonged drought conditions and in the recovery period. Modifications of H3K4me3 and H3K9ac were found to be positively correlated with the gene transcription level. Histone modifications of H3K4me3, H3K9ac indeed serve as a blueprint for stress memory. Transcription levels of stress-responsive genes for abscisic acid synthesis and signaling pathways were either maintained longer, even after the recovery period (example: *ZEP1*, *NCAD6*, *AP2/EREBP*, *NAC*), or some responsive genes stored the signal for a delayed response (example: *MADS4,15*) (Forestan et al., 2020).

Modifications of DNA methylation of genes, promoters, or transcription factors in response to drought stress have been detected in various plant species (Akhter et al., 2021; Czajka et al., 2021). Under drought stress conditions, plants exhibit dynamic and variable methylation levels, however the methylation changes are not always related to known transcriptome regulation associated with that stress. In mulberry plants, the overall methylation level of plants subjected to drought stress was 8.64% higher than that of well-watered ones (Li et al., 2020), while about 29% of DNA methylation processes were detected to be irreversible in rice plants exposed to drought stress (Wang et al., 2010). The degree, level and polymorphism of DNA methylation were different in wheat (Duan et al., 2020) or rice (Zheng et al., 2013; Wang et al., 2016) varieties differing in their response to water deficit. In general, drought increases the level of DNA methylation in non-adapted plants, however, if plants were previously adapted to the stress, the DNA methylation level decreased (Rendina González et al., 2018). Under drought conditions, hypermethylation events occur in the drought-susceptible genotypes while drought-tolerant genotypes present hypomethylation behavior (Gayacharan and Joel, 2013). DNA methylation can persist through some generations leading to transgenerational plasticity of the offspring (Herman and Sultan, 2016). Zheng et al. (2017) found that rice exposed to drought conditions had several stable methylation changes in stress-responsive genes which were passed on to progeny for multiple generations (Zheng et al., 2017).

The BRAHMA-type ATP-dependent chromatin remodeling factors (CHR12 or SW1/SNF2) play an essential regulating role in response to drought in *A. thaliana* (Han et al., 2012). Thousands of regulatory RNAs were identified in response to drought stress including miRNA, hc-siRNA, sRNA, and lncRNA-mediated regulation of gene expression and post-transcriptional modifications in several crops (Jha et al., 2020).

### 2.2 Salinity

High salinity causes ion toxicity and hyperosmotic stress, which inhibit plant development and productivity (Wani et al., 2020). Although the involvement of DNA methylation and different histone modification marks in regulating salt tolerance was demonstrated in various crops, the specific roles of DNA methylation in salt stress responses remain to be clarified (Liu and Lang, 2020). Salt stress induces, in different plant species, opposite effects on 5 mC (methylation or demethylation) of transcriptional regulators, to differentially modulate the downstream expression of salinity-related genes. In soybean and rice, salt stress induces 5 mC demethylation at the promoter of specific transporters, associated with a higher expression and with increased tolerance to salinity stress (Zhu et al., 2015; Zhang W. et al., 2020). In other cases, salinity stress induces an increase in 5 mC levels that may influence the expression of transporters or miRNA, thereby improving the salt tolerance (Ganie et al., 2016; Kumar et al., 2017). Most of the observed methylation/demethylation changes were stable after plant recovery, implying a possible establishment of a stress memory.

In salt-stressed rice, different DNA methylation patterns were identified in 14 zinc-finger-containing genes (Ahmad et al., 2019). Most methylation/demethylation changes were stable after recovery, implying a possible establishment of stress memory. In Foxtail millet (*Setaria italica* L.), a crop that is considered more tolerant to environmental stresses compared with other cereal crops, a strong decrease in DNA methylation levels was found in a salt-tolerant line when compared with a salt-sensitive variety under salt stress conditions. Promoter regions and coding sequences of several genes were hypomethylated including ABC transporters, WRKY transcription factors, serine-threonine protein phosphatases, and genes related to disease resistance and retrotransposon activation (Pandey et al., 2017). Methylation changes under salt stress were also observed in wheat. For example, the transcriptional level of the GAPC1 (Cytosolic glyceraldehyde-3-phosphate dehydrogenase) gene was induced under both osmotic and salinity stresses, accompanied by decreased methylation of CG and CHG cytosine residues in the promoter region of this gene. GAPC (Cytosolic glyceraldehyde-3-phosphate dehydrogenase) catalyzes a key reaction during glycolysis and was suggested to positively regulate stress responses in plants (Fei et al., 2017).

In maize seedlings, expansin genes were induced in response to salinity, leading to cell enlargement. Expansin-related genes showed elevated promoter H3K9ac levels accompanied by global accumulation of H3K9ac and H4K5ac under salt stress (Li Hui et al., 2014). Elevated levels of H3K9ac and H3K27ac marks were identified in the coding region of the peroxidase (POX) encoding gene in beet plants, which was transcriptionally activated by salt treatments. These marks were linked with high POX transcript abundance in both sugar beet (*Beta vulgaris* L.) and wild beet [*Beta vulgaris subsp. maritima* (L.) Arcang.], but the degree and the site of acetylation were different between the species and subspecies (Yolcu et al., 2016). Lastly, in alfalfa (*Medicago sativa* L.) alterations in the methylation status of the promoter region of the transcription factor MsMYB4 were detected following salinity stress. Activation of MsMYB4 was associated with an increased level of histone H3K4 trimethylation and H3K9 acetylation in it’s the corresponding promoter (Dong et al., 2020).

### 2.3 Cold/High Temperature

Since temperature is a key factor governing plants/crops growth and development, either high or low temperatures limit their productivity and yield. The link between epigenetic processes and plant responses to non-optimal temperature conditions was demonstrated on the molecular, biochemical, and cellular levels (Ueda and Seki, 2020). Recent studies show that the expression of 29 genes in a cold-tolerant rice line was altered under cold stress, in correlation with changes in DNA methylation, mostly at promoter regions (Guo et al., 2019). Similarly, even histone modifications are involved in cold/heat stress response through gene expression tuning (Bannister and Kouzarides, 2011; Kim et al., 2015). This is due to a large number of DNase I hypersensitive sites (DHSs) induced by cold stress, translating this event into enhanced chromatin accessibility.

For instance, in plants is known that the euchromatin mark H3K4me3, which indicates the tri-methylation at the 4th lysine residue of the histone H3 protein, is commonly associated with the activation of transcription of nearby genes (Zhang et al., 2009). In contrast, H3K27me3, indicating the tri-methylation of lysine 27 on histone H3 protein, is associated with one of the major gene silencing systems in plants (Zhang et al., 2007). Genome-wide distributions of these histone modifications and their association with gene expression have been well-documented in several plant species as *A. thaliana* and potato (Zhou et al., 2010). The cold stress may induce the H3K27me3 deposition, which, in turn, has been demonstrated to be involved, for instance, in Flowering Locus C downregulation.

However, several cases of bivalent histone modifications of H3K4me3 and H3K27me3 are known and associated with cold stress related genes (about 6,500). In particular, Zeng et al. (2019) demonstrated that active genes (transcribed in both conditions) displayed enhanced chromatin accessibility upon cold storage. Upregulated genes, associated with this bivalent mark, were enriched in functions and related to the stress response, while the downregulated genes were involved in the developmental processes. The authors hypothesized that the bivalent H3K4me3-H3K27me3 mark represents, in potato tubers, a distinct chromatin environment with greater accessibility, which might facilitate the access of regulatory proteins required for gene upregulation or downregulation in response to cold stress.

Regarding heat stress, CHH methylation patterns differed between two rice lines showing different levels of heat tolerance (He et al., 2020). In barley (*Hordeum vulgare* L.), increasing air temperature by 3°C led to increased levels and altering DNA methylation patterns while in cotton (*Gossypium hirsutum* L.) prolonged heat stress led to methylation changes in the promoter of anther-expressed genes. These changes promoted a series of redox processes to support a different development program under stress conditions (Zhang X. et al., 2020).

### 2.4 Visible and Ultraviolet Light

Light is essential for photosynthesis and also for conveying information on environmental conditions such as wavelength composition, direction, intensity, and photoperiod. Plant photoreceptors are specialized in perceiving light stimuli ranging from ultraviolet (UV) to visible and far-red (FR) irradiation that induce downstream signaling events including major transcriptional reprogramming. There is increasing evidence of how light triggers changes in chromatin compaction, nuclear morphology as well as influencing histone modifications and gene repositioning (Perrella et al., 2020). Plant photoreceptors and downstream signaling components interact and modulate the action of chromatin remodeling enzymes and transcriptional regulators that confer light-induced chromatin changes through the deposition of epigenetic marks. Early studies revealed that histone acetylation is associated with the induction of gene expression in response to light. These initial observations were further verified by physiological and molecular experiments on mutants of histone acetyltransferase (GCN5, HAF2) and deacetylase enzymes (HDA15, HDA6) (Bourbousse et al., 2020; Perrella et al., 2020). Furthermore, the role of histone H2B mono-ubiquitination was linked to light-induced activation of gene expression in light-grown *A. thaliana* seedlings by facilitating the activity of RNA Polymerase II (Bourbousse et al., 2012). Changes in histone methylation levels have been also associated with shade avoidance responses, which are triggered by a decrease in the R/FR ratio due to canopy coverage. Shade induces growth-promoting genes leading to the elongation of hypocotyls, stems, and petioles in search of light (Martínez-García and Moreno-Romero, 2020). An increase in H3K4me3 and H3K36me3 levels and recruitment of the histone methylation reader MRG2 on growth-promoting loci such as *YUCCA8* has been reported to mediate shade-induced physiological responses in *A. thaliana*.

Studies in *A. thaliana* and maize have shown that UV-B induces an increase in histone H3 and H4 acetylation levels (Casati et al., 2006; Casati et al., 2008; Campi et al., 2012). Members of multiple histone acetyltransferase families, such as HAM1, HAM2, HAC1, HAG3, and HAF1, have also been shown to regulate different UV-B signaling responses (Fina et al., 2017). Furthermore, UV-B can negatively regulate the transcript levels of the Polycomb Repressive Complex two components MSII and CURLY LEAF that control H3K27me3 deposition on the flowering regulating loci *MIR156* and *FLC* (Dotto et al., 2018). As a result, UV-B leads to delayed flowering in *A. thaliana* (Dotto et al., 2018). The UV-B receptor UVR8 also plays a role in controlling a UV-B-dependent increase in the acetylation status of histone H3 lysine K9 and K14 on target genes (Velanis et al., 2016). Furthermore, UVR8 regulates DNA methylation by directly associating and inhibiting DRM2 (Jiang et al., 2021). In addition to regulating plant development, UV-irradiation induces DNA damage responses leading to changes in chromatin and epigenome dynamics. However, the exact molecular interplay among DNA-damage repair and acclimation responses to high light and temperature requires further investigation (Molinier, 2017).

### 2.5 Heavy Metals and Metalloids

Among the abiotic stresses affecting plant wellness, heavy metal (HM) contamination represents a serious threat also to humans and animals. In plants, exposure to excessive amounts of both essential and non-essential HMs induced toxic effects, activating a broad array of alterations (Edelstein and Ben-Hur, 2018). In this relation, many recent studies suggest that climate change has both a direct and indirect effect on HM leaching and bioavailability (Fan and Shibata, 2015; Xia et al., 2016). The raising temperatures and the related increase in atmospheric CO_2_ levels, which indirectly increase chemical weathering due to both temperature and lower pH, lead to the release of metals in the earth’s crust and soils (Whitehead et al., 2009; Benítez-Gilabert et al., 2010). On the other hand, precipitation has an impact on surface runoff, river discharge, and thus indirectly on river water quality. Surface runoff is an important carrier of contaminants from the surrounding land (brownfields) to the receiving surface water. The consequences of these effects are leading to degradation in water and sediment quality that could have negative impact on the ecosystems. Growing evidence highlights important roles in plant adaptation to highly HM and metalloids contaminated environments of epigenetic variations, often responsible for modulating gene expression (Cicatelli et al., 2014; Kumar, 2018). This phenomenon is mediated by a complex interplay among different molecular factors: changes in DNA methylation patterns, histone modifications and chromatin remodeling (Dutta et al., 2018). A recent study suggests that DNA demethylation is one of the molecular strategies adopted by *Arundo donax* L. plants to counteract the stress caused by soil arsenic pollution (Guarino et al., 2020).

Many HM-related RNAs have been identified and several findings are indicating their important role as trans-acting epigenetic signals, involved in specific gene regulatory networks activated in response to HM stress in plants (Ding et al., 2020). For instance, aluminum can induce a hypomethylation of the NtGPDL gene in tobacco. This carries the information for an aluminum stress-activated glycerophosphodiesterase (Choi and Sano, 2007). In wheat, hypermethylation was obtained with the highest concentrations of aluminum and hypomethylation with the lowest ones (Hossein Pour, 2019). Gallo-Franco et al. (2020) calculated the methylation level of 26 genes from the IR64, Nipponbare, and Pokkali varieties of rice plants using data in the scientific literature and the Rice SNP Seek database. All three varieties were hypermethylated with the highest levels in the Nipponbare variety, and the ART1 and STAR1 genes were differently methylated. These genes encode and regulate the transcription of transmembrane proteins useful for aluminum detoxification. Similarly, Gullì et al. (2018) showed that a specimen of *Noccaea caerulescens* grown in an area with nickel-rich soil showed a genome that was more methylated than the control. The overexpressed genes were the *MET1* DNA methyltransferase, the *HDA8* histone deacetylase, and the *DRM2* DNA methyltransferase involved in RdDM. All three were upregulated from 3 to 16-fold.

### 2.6 Nanomaterials

A large number of new materials is produced for human use. Their environmental dispersal under climate change conditions has led to increased pollution and risk to the health of plants, animals, and humans. Waste dispersed in the environment undergoes degradation processes that cause the dispersion of nanoparticles and pollutants with varying toxicological characteristics (Nejdl et al., 2020).

Nanomaterials are particles smaller than 100 nm that can be of natural or artificial origin. The first category includes those formed by natural processes such as volcanic activities and air particles while the second includes those synthesized for biomedical and industrial purposes. Studying the effects of nanomaterials on the plants is therefore critical to understanding the impact of the pollutant on the ecosystem. Nanoplastics as new pollutants can get adsorbed by plants. The main route of plant intoxication is the root route with the uptake of nanoparticles from the polluted soil (Deng et al., 2014). Roots can take up nanoparticles symplastically or apoplastically. In the former, internalization occurs *via* endocytosis or *via* aquaporins, the number of which affects the uptake (Rico et al., 2011). In the latter, nanoparticles cross the spaces between cells, if their size is smaller (Zhao et al., 2012). Absorption can also occur through leaves but to lesser extent (Deng et al., 2014). Other general effects include the production of ROS resulting in increased lipid peroxidation, DNA degradation, and cell death (Tarrahi et al., 2021). So far, few studies have addressed the possible involvement of epigenetic processes in the response of plants to these types of stress. However, the observation that ROS are part of the cell response to nanomaterials could suggest that ROS-mediated epigenetic regulation is also involved.

Carbon-based nanomaterials are to date used for a lot of industrial purposes and studied for their nanotoxicology in plants (Marmiroli and White, 2016). It has been shown how carbon nanotubes can pierce the root walls of plants and enter both apoplastically and symplastically (Tripathi et al., 2017). Once adsorbed, they can reach organelles such as mitochondria and chloroplasts and especially in the nucleus of plant cells (Jordan et al., 2018). In *Allium cepa* L. several variations were observed depending on the concentration of the Multi-Walled Nanomaterials (MWNM) used. The cutting sequence of the restriction enzyme *Hpa* II was found to be hypomethylated at low concentration and hypermethylated at high concentration (Ghosh et al., 2015). Single-wall and multi-wall carbon nanotubes were found to promote rice root growth, by eliciting similar molecular pathways and epigenetic regulation (Yan et al., 2016).

Contrasting results were obtained for silver nanoparticles (AgNPs), a large family of materials used from the home appliance industry to the cosmetic industry. AgNPs formulation Argovit™ showed no cyto- or genotoxic damage or epigenetic effects in *A. cepa* (Casillas-Figueroa et al., 2020). However, in *A. thaliana*, the evaluated concentrations resulted in increased expression of genes involved in glutathione biosynthesis, glutathione S- transferase, and glutathione reductase (Nair and Chung, 2014).

## 3 From Alphabet to Syntax – Recommendations for the Future

Enormous progress has been made in understanding the role of epigenetic regulation in crop response to different stresses. However, in order to make step and translate an “epigenetic alphabet” into “epigenetic syntax” and evolve from “experimental” to “classical” methodology in crop breeding, epigenetics needs to overcome four main challenges:

(1) Need for improved experimental procedures, especially in sequencing technology (longer reads, deeper single-cell sequencing, more efficient sample preparation kits, sequencing portable device improvements);
(2) Need for improved workflows of data analysis, as epigenomic data are currently dispersed, obtained with different methodologies and approaches. Indeed, there is an urgent need of defining and delivering approved methodological standards for both wet-lab and *in silico* analysis. The first steps are made in this direction with solutions offered to improve data workflow systems with cloud services and use of open data for bioinformatics research (Rezaul Karim et al., 2018) and development of standardized workflows for epigenetic data such as ARPEGGIO (Milosavljevic et al., 2021);
(3) Need for enhanced knowledge on crop species at all epigenetic levels as well as interactions between epigenetic machinery and other TF or DNA binding proteins to gain insight into the interactions between epigenome and changes in DNA sequences. Future directions to hasten application of epigenetic modifications in crop breeding strategies for specific agronomical traits have been proposed by several authors (Gallusci et al., 2017; Varotto et al., 2020; Kakoulidou et al., 2021), and need to be applied on wider scale in order to transfer knowledge from model plants to crops;
(4) Need to better integrate epigenomic data with other “omics” data, since epigenomic data are difficult to match with data obtained at other “omics” levels. This highlights the need for agreeing which standards and workflows have to be followed in experiments comprising different “omics” analyses. Hence, constructive and methodological guidelines on how to perform multi-omics data integration (MOI) in plants are needed. Studies of Jamil et al. (2020) who propose three levels of MOI—element-based, pathway-based and mathematical-based integration and Grabowski and Rappsilber (2019) who provide practical guide on how to move from data to insight while using easily accessible data sources, could be good models for future work in “omics” data integration.

Overcoming above-mentioned challenges will facilitate: *i*) elucidation of the role of other mechanisms, besides chromatin-based mechanisms, in somatic and inter-generational stress memory and understanding if there is a universal mechanism of stress memory or if different cases of stress memory are modulated in a different way; *ii*) demonstrating if targeted, gene-specific epigenome or epi-transcriptome modifications anticipated responses to stresses, that will allow the identification of key regulatory mechanisms for tailored responses to the new challenges driven by climate change; *iii*) understanding how epigenetic changes can produce new stable phenotypes in a few generations, allowing the plant survival in their natural habitats; *iv*) clarification of the role of chromatin structure modifications in hypersensitivity reaction, contributing to plasticity and plant adaptation in a world context of climate change; *v*) clarification of the role of RdDM machinery, together with other DNA methylation mechanisms targeting and often silencing repetitive elements, highly represented in the plant genome and *vi*) identification of the difference between correlation and causality, that is if a chromatin regulator is required for a particular stress response, it does not necessarily imply that it modulates the stress response, as it may be a passive response affecting gene expression, rather than being an endogenous regulation of the process. Consequently, silencing of a chromatin regulator may cause a stress response not through the action of stress-responsive genes, but indirectly due to phenotypic, metabolic, and developmental modifications.

The interdisciplinary effort of scientists involved in plant biology and crop improvement in resolving the above-mentioned issues and gaining new insights into epigenetics mechanisms involved in plant stress response should pave the way for further understanding of an epigenetic alphabet of plants and its translation into epigenetic syntaxes for further exploitation of epigenetic variation in crop breeding for climate resilience.
